# What causes failure of fixed orthodontic retention? – systematic review and meta-analysis of clinical studies

**DOI:** 10.1186/s13005-021-00281-3

**Published:** 2021-07-24

**Authors:** Maciej Jedliński, Katarzyna Grocholewicz, Marta Mazur, Joanna Janiszewska-Olszowska

**Affiliations:** 1 Department of Interdisciplinary Dentistry, Pomeranian Medical University in Szczecin, 70-111 Szczecin, Poland; 2 Department of Dental and Maxillofacial Sciences, Sapienza University of Rome, 00161 Rome, Italy

**Keywords:** Fixed retainer, Fiber splint, Breakage, Debonding, Relapse

## Abstract

**Background:**

Orthodontic retention aims to maintain optimal teeth positions after active treatment. The stability is affected by numerous factors, including patients’ individual features, thus retention should be adjusted in the most optimal way. Bonding a retainer makes retention less dependent on patient’s compliance.

**Questions arise:**

What wire or fiber splint type provides the best treatment stability? What materials should be used to bond the wire or fiber splint? Should be the bonding procedure be direct or indirect? The aim of the study is to assess and synthesize available controlled trials investigating failures of fixed retainers.

**Methods:**

Literature searches of free text and MeSH terms were performed in Scopus, Web of Science, Embase and PubMed Central in order to find studies, referring to failures of fixed retention (12th February 2021). The keywords were: (“orthodontic retainers AND failure AND wire”). The framework of this systematic review according to PICO was: Population: orthodontic patients; Intervention: fixed orthodontic retainer bonding; Comparison: Different protocols of fixed orthodontic retention applied; Outcomes: failure rate, survival rate. Three different specific scales from the Cochrane Collaboration Handbook were used, according to each study type. Additionally, a meta-analysis was conducted to compare the effectiveness of retention using fiber reinforced composite and multistranded steel wire.

**Results:**

The search identified 177 potential articles: 114 from PubMed, 41 from Scopus, 20 from Web of Science and 2 from Embase. After excluding studies inconsistent with selection criteria, 21 studies were included and subjected to qualitative analysis. The main outcome investigated was failure rate. This systematic review has some potential limitations due to the heterogeneity of design between included studies.

**Conclusions:**

No retainer is proved to guarantee a perfect stability of dental alignment. The retainer should be bonded to all adherent teeth, preferably with additional use of bonding resin. No wire or fiber splint present superior characteristics concerning failure rate. Fiber reinforced composite retention is more sensitive to operator skills, and with imperfect bonding technique, failure rate is much higher. During the first 6 months after bonding retainer the patient should be under frequent control. The study protocol was registered in PROSPERO database with the number CRD42021233406.

**Supplementary Information:**

The online version contains supplementary material available at 10.1186/s13005-021-00281-3.

## Background

Orthodontic retention is defined as maintaining optimal aesthetic and functional teeth positions after cessation of active orthodontic treatment. It should be recognized as an integral phase of orthodontic treatment [1]. Two forms of retention can be distinguished: fixed - in the form of appliance attached to the lingual tooth surface, or removable- in the form of acrylic plates or transparent thermoformed splints [2].

The stability of orthodontic treatment is affected by numerous factors, including type of initial malocclusion, age, gender, pathology of the surrounding soft tissues, patient compliance and retention protocol applied [3]. A potential relapse may be due to: lasting remodeling periodontal tissues, muscular imbalance or changes produced by growth and ageing [4]. Tooth movement resulting from ageing occurs in all subjects, disregarding the positive or negative history of orthodontic treatment. Little et al. [5] in their long-term observational studies in 1988 found that 90% of cases relapse within 10–20 years after the end of retention. The tendency to relapse or post-treatment undesired tooth movement varies in individual cases [4].

Since orthodontists have no influence on the patient’s individual features, the protocol of retention phase should be adjusted in the most optimal way. The results of the latest survey studies have shown that European orthodontists are much more willing to use permanent retention devices, especially in the area of lower anterior teeth [6, 7]. The greatest approval of specialists enjoys retention carried out simultaneously with a removable retention device and a fixed retention device [6, 7].

Nowadays people pay a great attention to their appearance, thus the demand for esthetic orthodontic treatment is increasing. Patients place great emphasis on the stability of treatment results. Treatment involves not only physical but also emotional effort for patient [8, 9] thus a possible relapse causes dissatisfaction. For the best compliance, the patient should apply for regular appointments several times a year during retention phase [10]. A special information campaign “Hold that smile” was held by the British Orthodontic Society, to educate patients on the importance of the retention phase of orthodontic treatment, and enhance a better cooperation [11]. Nevertheless, the use of removable retainers always depends most on the patient’s self-discipline, whereas the doctor’s influence remains limited.

Bonding a fixed retainer makes retention less dependent on patient’s compliance. Questions arise concerning the wire: What wire or fiber splint type provides the best treatment stability? What materials should be used to bond the wire or fiber splint to tooth surface? What should be the procedure of bonding fixed retention (direct or in-direct)? Since opinions on this subject vary, the authors have tried to systemize the current knowledge about bonding fixed retention in order to try to create clinical recommendations.

## Aim of the study

The aim of the study was to assess and synthesize available controlled trials investigating the failures of fixed retainers.

## Materials and methods

### Search strategy

This systematic review was conducted according to the PRISMA statement [12], the PRISMA reporting guidelines [13, 14] and the guidelines from the Cochrane Handbook for Systematic Reviews of Interventions [15]. Literature searches of free text and MeSH terms were performed in Scopus, Web of Science, Embase and PubMed Central search engines in order to find studies, exploring the topic of bonding fixed retention in terms of failures (12th February 2021). All searching was performed using a combination of subject headings and free-text terms: the final search strategy was determined through several pre-searches. The keywords used in the search strategy were as follows: (“orthodontic retainers AND failure AND wire”). The study protocol was registered after the screening stage in PROSPERO database with the number CRD42021233406. The framework of this systematic review according to PICO [16] was: Population: orthodontic patients; Intervention: fixed orthodontic retainer bonding; Comparison: Different protocols of fixed orthodontic retention applied; Outcomes: failure rate, survival rate. The included articles discuss the choice of retention wire, the type of composite used for bonding, and try to answer why failure occurs when using fixed retention.

### Eligibility criteria

The following inclusion criteria were applied for this systematic review:

- Type of study: randomized clinical trials (RCTs), cohort studies, case – control studies (CCSs), retrospective studies on fixed retainers efficacy.
- Results of the study: Fixed retention failure defined as debonding or undesired tooth movement.
- Object of the study: a) comparison of the efficacy of wires or fiber glass splints bonded with same procedure and using the same material or b) comparison of efficacy of bonding materials with the same type of procedure and the same type of wire
- Subject of the study: human subjects

The following exclusion criteria were applied: incomplete studies, in-vitro studies, studies in which concurrent procedures were applied (as fibrotomy), lack of effective statistical analysis; papers not related to fixed retention failure, studies not written in English.

### Data extraction

Titles and abstracts were independently selected by two authors (MJ and KG), following the inclusion criteria. The full text of each identified article was then analyzed to verify, whether it was suitable for inclusion. Whenever disagreement occurred, it has been resolved by discussion with the third author (JJ) by creating a spreadsheet in order to compare them through according to the Cochrane Collaboration guidelines [15]. Authorship, year of publication, type of each eligible study and its relevance regarding to the use of fixed orthodontic retention were extracted by one author (JJ) and examined by another author (MJ).

### Quality assessment

According to the PRISMA statements the evaluation of methodological quality gives an indication of the strength of evidence provided by the study because methodological flaws can result in biases [12].

The quality assessment of RCTs was performed using the revised tool for assessing risk of bias in randomized trials (RoB 2) [17]. This tool assesses the possible risk of bias by evaluating five characteristics: sequence generation, allocation concealment, blinding to personnel, blinding to outcome analysis and incomplete outcome bias in accordance with the Cochrane guidelines. There are three possible grades for each characteristic: low RoB – meaning no bias, or if present, rather unlikely to alter the results significantly, some concerns – meaning risk of bias that raises some doubt about the results and high RoB - bias may alter the results significantly [18]. If other type of bias occurred, it has been described more specifically. What is more, the quality assessment of CCSs and retrospective studies was performed using Newcastle – Ottawa Scale for Case-control studies [19]. To perform the evaluation of eventual risk of bias in cohort studies the Newcastle-Ottawa Quality Assessment Form for Cohort Studies has been employed [20]. The quality assessment of all included studies in both scales of Newcastle-Ottawa Assessment forms is based on object selection, comparability and ensuring undisturbed overlap of processes leading to the outcome. The possible quality assessment score ranged from 0 to 9 points, with the higher score indicating the better quality of given study. For each characteristic evaluated, one point was given. The study could receive 4 points for the ideal object selection, 2 points for the ideal comparability and 3 points for the ideal determination of the exposure and its evaluation.

### Meta-analysis

Meta-analysis was performed using random-effect model via metafor R package, [21] with LRR (Log Risk Ratio) and 95% confidence intervals (95% CI) being calculated as effect estimates. LRR is relatively insensitive to differences in baseline risk [22]. Heterogeneity was assessed quantitatively using I2-statistics and Cochran’s Q [23]. Publication bias was estimated using funnel plot.

## Results

### Search results

**Fig. 1:**
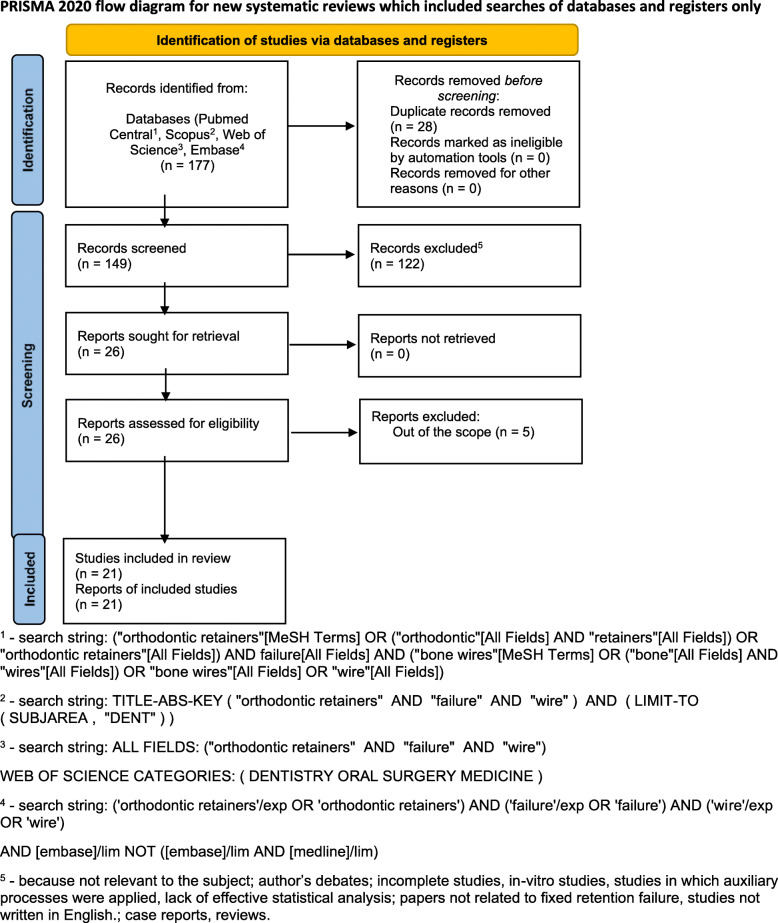
Prisma 2020 Flow Diagram representing study selection process

The search strategy identified 177 potential articles: 114 from PubMed Central, 41 from Scopus, 20 from Web of science and 2 from Embase. After removal of duplicates, 149 articles were analyzed. Subsequently, 112 papers were excluded because they did not meet the inclusion criteria. Of the remaining 26 papers, 5 were excluded because they were not relevant to the subject of the study. They focused mainly on other phenomena - such as influence of fixed retention on periodontal tissues or did not meet inclusion criteria, because of type of the study. The remaining 21 papers were included in the qualitative synthesis. Table 1 summarizes the characteristics of each of the included studies. Prisma 2020 Flow Diagram representing study selection process has been presented in Fig. 1.

**Table 1:** Characteristics of included studies

Author and year of publication	Type of study	Study objective	Number of subjects	Comparison made	Outcome measured	Results
**Bovali et al. 2014** [24]	RCCT	Comparison of the bonding time and number of failures between direct and indirect bonding procedure	64 patients (35 W, 29 M)32 patients per group	Direct and indirect bonding technique using a round 0.0215 in multistrand stainless steel wire (Penta One; Masel, Philadelphia, Pa	The time needed for bonding mandibular lingual retainers and the number of failures at any time within 200 days time (checked 1st,2nd,4th and 6th month)	The indirect bonding technique resulted in a significantly shorter chairtime (321 ± 31 s) than the direct technique (401 ± 40 s). The log-rank test showed no statistically significant difference between the survival rates of the indirect and direct bonding techniques (*P* = 0.35). The learning curve applied both to chairtime and failures.
**Gunay et al. 2016** [25]	RCCT	Comparison of the failure rate of fixed orthodontic retention between 2 different wires	120 patients60 patients per group(83 W, 37 M)	0.0175-in 6-strand stainless steel wire (Ortho Technology, Lutz, Fla)Vs.0.0195-in dead-soft coaxial wire (Respond; Ormco, Orange, Calif)	Debonding rate within 12 month period (every 3 months)	The bond failure rates were 13.2% for the 0.0175-in 6-strand stainless steel wire and 18.9% for the 0.0195-in dead-soft wire. Mandibular irregularity index increased with time in both groups.
**Bolla et al. 2011** [26]	RCCT	Comparison of the failure rate and breakage rate of fixed orthodontic retention between 2 different wires	85 patients (56 W 29 M) with 98 retainers (32 maxillary, 66 mandibular)	Glass fiberVs.0.0175 multistranded stainless steel wire	Debonding rate and breakage rate within 6 years period (every 3 months in 1st year, 6 months in following years)	The failure rates were comparable in the GFR and 0.0175 multistranded groups in terms of detachment and breakage. However, the overall failure rate is quite high. Detachment in the maxillary arch occurred in 3/14 glass fiber retainers (21.42%) and 4/18 SS wire retainers (22.22%); in the mandibular arch detachment was recorded 4/34 glass fiber retainers (11.76%) and in 5/32 SS wire retainers (15.62%).Interproximal wire breakage in the maxillary arch was observed in only 1/14 glass fiber retainer (7.14%) and in 3/14 MST retainers (16.66%); in the mandibular arch, in 3/34 glass fiber retainers (8.82%) and in 5/32 SS wire retainers (15.62%). It can be concluded that in both groups, detachment happens equally often, and that statistically it is a more frequent cause of failure. Wire breakage was less common in the glass fiber group, but the differences are not significant.
**Årtun et al. 1997** [27]	RCCT	Comparison of failure rate and the ability of maintain alignment in the anterior segment of the jaw.	49 patients in irregular groups	Thick plain wire bonded only to canines VsThick spiral wire bonded only to canines VsThin spiral wire bonded to incisors and canines VsRemovable retainers	Failure rate, level of plaque and calculus accumulation (Gingival Index, Calculus Index, Plaque Index, Loss of attachment) within 3 years period	A total of 8/35 (22,9%) failed, one during the first year, one during the second year, and six during the third year. Failures occurred in 4/13 retainers made of thick spiral wire bonded only to the canines (30.8%), 1/11 made of thick plain wire bonded only to the canines and 3/11 made of thin, flexible spiral wire bonded to each tooth (27.3%). Two of the 14 removable retainers (14.3%) were lost, what was also considered as failure. Plaque was observed more frequently gingivally to the wire. No significant differences in plaque nor calculus accumulation were found between the wires.The smallest differences in the mandibular irregularity index were observed in patients with thin spiral wire bonded to all teeth, and the largest in patients with thick plain wire bonded only to canines and in patients with removable retainers.
**Nagani et al. 2020** [28]	RCT	Comparison of failure rate and evaluation of failure pattern in 2 types of mandibular canine-canine bonded retainers.	52 patients (8 W, 44 M), 26 patients per group The adhesion to single dental element was considered separately	Fiber reinforced composite retainers (INOD, U.P. Fiber Splint, 2 mm)Vs.Multistranded stainless steel wire retainers (All Star Orthodontics, 0.0175 in.)	Failure rate within 12 months (checked monthly)	The bond failure rates were 42.94% for patients with fiber reinforced composite retainer and 31.41% for multistranded SS wire. Hence, total number of bond failures in both groups were 37.17%. The difference between those groups were statistically significant. Adhesive failure (no retained resin on enamel surface) is the most common type of bond failure observed with both groups of fixed lingual retainers. (23% of all failures). According to their finding, multistranded SS wire is superior option to fiber splin.
**Bazargani et al. 2012** [29]	RCT	Evaluation of the use the effect of liquid resin on the survival of fixed lingual retainers and to evaluate the incidence of calculus accumulation and discoloration adjacent to the lingual retainers.	52 patients (26 W, 26 M) dived into 2 equal groups	0.0195-in. multistranded Penta-one wire (Masel Orthodontics, Bristol, Penn) bonded with the use of Optibond FL resign and Tetric Evo flow composite Vs.Wire + Tetric Evo flow composite only	Failure rate, calculus accumulation and eventual discoloration due to retainers presence within 2 years (checked monthly)	In the resin group, the failure within 2 years occurred once in one patient only (4%), while at the composite-wire interface. In the nonresin group, the incidence of retainer failure occurred once in seven different patients (27%), from one or several teeth, and all at the enamel-composite interface. The incidence of calculus accumulation (4 to 31%) and discoloration adjacent to the composite pads (0 to 69%) was much more higher in the nonresin group.
**Salehi et al. 2013** [30]	RCT	Comparison of failure rate and survival rate of two types of fixed orthodontic retainer	142 patients (83 W, 59 M) divided into unequal groups (68–74)	Polyethylene woven ribbon (Ribbond, Seattle, WA, USA) Vs.0.0175-in flexible spiral wire (Respond, Ormco, Glendora, CA, USA).	Survival and rate within 18 month period (checked monthly)	There was no statistically significant differencebetween the two types of retainers in the maxillary or the mandibular arches in terms of survival rate. One-tooth failure was the most frequent failure of the two retainer types. Among all the retainers in all experimental groups, only in one case the multi-stranded retainer was completely detached in the maxilla. The most frequent type of failure in the multi-stranded group was retainer loosening, both in the maxilla (22/27 (81.48%)) and in the mandible (27/28 (96.42%)). In the ribbon retainer group, the most frequent type of failure was retainer fracture in the maxilla (30/34 (88.23%)) and retainer loosening in the mandible (19/29 (65.51%)). This study showed that the mean survival time and the rates of broken or detached ribbon retainers and multistranded retainers are comparable. Ribbon were more prone to breakage, while spiral wires debonded more often.
**Arash et al. 2020** [31]	RCT	Comparison of failure rate of two types of fixed orthodontic retainer	260 patients (161 W, 99 M) divided into unequal groups (138–122)	0.0175 stainless steel twisted wire (G&H Orthodontics, USA)Vs.single-strand ribbon titanium lingual retainer wire (Retainium, Reliance orthodontics, USA)	Failure rate and timing of failure within 2 years (checked monthly)	Failure rates in terms of detachments in all groups seemto have occurred at the enamel junction which is clinicallyobserved the bulk of detached composite, and it was25 in twisted retainer group (18.1%) and was 10 in ribbonretainer group (8.9%), what points out ribbon titanium retainer as more reliable. The average duration of success was ca. 23,5 months for both twisted wire and ribbon wire, what shows, that failure of retention was not frequent phenomenon.
**Scribante et al. 2011** [32]	RCT	Comparison of failure rate of two types of fixed orthodontic retainer in mandibular arch	34 patients (9 W, 25 M)	A multistrand stainless steel wire (Ortosmail Krugg, Milan, Italy)Vs.a polyethylene ribbon-reinforced resin composite (InFibra TPItalia, Gorle, Italy)	Survival rate, patients satisfaction measured with VAS scale within 12 months (30, 60, 120, 180, 360 days to evaluate detachments)	The percentage of detachment was 22,54% for stainless steel wire teeth to 14,45% for polyethylene ribbon-reinforced resin retainer. The patients with multistrandedstainless steel wire expressed a mean value of satisfaction of 8.24, whereas patients with polyethylene fiber reinforced resin retainer for lingual retention expressed a mean value of satisfaction of 9.73. The failure rate did not statistically differ between two types of reteiners, however polyethylene ribbon-reinforced resin composite is considered as more aesthetic for patients.
**Rose et al. 2002** [33]	RCT	Comparison of survival of multistranded stainless steel wire and a direct-bonded polyethylene ribbon-reinforced resin composite	20 patients (8 W, 12 M)	The polyethylene woven ribbon (Ribbond) Vs. the multistranded steel wire (Respond, 0.0175 in., Ormco)	Survival rate, distribution of failures in time within 24 months period (checked monthly)	The median survival time of the Ribbond reteiners was 15.8 months (standard error = 3.6 months). Fifty percent of the retainers were still in place after 24 months. The most frequent kind of failure was a loosening between the Ribbond and theComposite. The rate of retainer loosening was lower in the multistranded retainer group. The median survival time was 23.9 months. Among those 10 retainers, only one became loose within the study period; the remaining ones were still in place at 24 months.
**Gelin et al. 2020** [34]	RCT	Comparison of effectiveness of CAD/CAM customized nitinol retainers with standard stainless-steel fixed retainers	61 patients (43 W 18 M) Groups were designed as equal – one patient dropped out	Rectangular 0.014 × 0.014 in memory shape customized CAD/CAM nitinol retainer (Memotain™; CA Digital GmbH, Mettmann, Germany)Vs.Round 0.0175′ 6-strand twisted stainless-steel wire retainer (Supra-FlexTM; RMO Europe, Illkirch-Graffenstaden, France)	Failure rate, changes in intercanine width, interpremolar width, anterior arch length, total arch length, Gingival index, Plaque Index with other periodontal measurements within 12 months period. (checked monthly)VAS Scale was used to measure patients satisfaction.Little Index measured at the beginning, after 6 and 12 months and IMP angle were measured at the begging and in the end of treatment	The average number of debonding per patientshowed no significant difference from during the study between the two groups. The type ofdebonding (adhesive-enamel interface or wire-compositeinterface) was found also similar. The level of satisfaction in terms of the final result was the same in the two groups. Similar to the level of discomfort for the tongue. The changes of value of Little Index did not significantly differ between the two groups. Dental stability parameter measurements in the control and test groups also did not show any significant difference. The overall periodontal parameters also remained unchanged from baseline until the end of the study in each group, with exception of Gingival Index, which was higher in test group. From begging to the end of the study, theIMPA and inter-incisor angles remained stable betweenthe two groups.
**Kartal et al. 2020** [35]	RCT	Comparison of effectiveness of CAD/CAM customized nitinol retainers with standard five-stranded steel retainers	52 patients (32 W, 20 M) divided in 2 groups of 26 patients	Rectangular 0.014 × 0.014 in memory shape customized CAD/CAM nitinol retainer (MemotainTM; CA Digital GmbH, Mettmann, Germany)Vs.0.0215′ five-stranded retainers (GC Orthodontics Inc., Alsip, IL, USA)	Failure rate, survival rate plaque index, gingival index, BoP, Probing depth, marginal recession within 6 months period (checked monthly)	No significant difference was observed for plaque index, gingival index scores, marginal recession, blooding on probing and probing depth between the groups on any visit during the follow - up. On the other hand, significant differences were observed within both groups for plaque index scores and probing depth obtained at different appointments during the 6-month follow-up period. No difference was observed for failure rate per tooth scores between the groups during any evaluation. All failures occurred due to debonding between the adhesive–enamel and none of the retainer wires were completely detached, deformed or broken. The survival rates of the retainer wires were 77% for the Memotain and 73% for the five-stranded group.
**Scribante et al. 2020** [36]	RCT	Comparison of failure rate of multistrand stainless steel wire attached with different composites	100 patients divided into equal groups	Use of composite resin Transbond XT (3 M, St. Paul, MN, USA)VsUse of Filtek Supreme XTE flowable nanocomposite (3 M, St. Paul, MN, USA),	Failure rate and survival failure rate within 24 months (checked monthly)	For both the upper and lower arch, as well as if considering them overall, higher total failures were found in retainers attached with flowable nanocomposite. The lower teeth reported a higher failure rate to the upper ones (13.33% vs. 10.67%). The detachment was not location- specific (none of the teeth come loose more often).
**Sfondrini et al. 2014** [37]	RCT	Comparison of failure rate of a resin composite retainer reinforced with glass fibers with a multistranded stainless steel wire	87 patients (52 W,35 M) 47 flexible spiral wire and 40 FRCs splints	Silanised-treated glass fibers (Everstick Ortho, Stick Tech ltd, Turku, Finland)Vs0,0175″ flexible spiral wire (Ortosmail, Krugg spa, Milan, Italy)	Failure rate and survival failure rate within 12 months(checked monthly)	17.73% (N of teeth = 47) for flexible spiral wires and 11.25% (N of teeth = 27) for glass fiber-reinforced resin retainers. No significant differences were found.
**Sobouti et al. 2016** [38]	RCT	Comparison of effectiveness of twisted wire fixed retainer versus spiral wire and fiber-reinforced composite retainers	128 patients (68 W, 60 M) in unequal groups due to expulsion from examination by exclusion criteria	0,0175″ flexible spiral wire (Ortosmail, Krugg spa, Milan, Italy)VsSilanised-treated glass fibers (Everstick Ortho, Stick Tech ltd, Turku, Finland)VsManually fabricated two twisted 0.009-in dead soft wires [6 rounds per 10 mm] (3 M Unitek, Monrovia, CA, USA)	Failure rate, Survival rate within 24 months	The average duration of success was approximately 21 months. Marginally significant difference was detected in the survival rates between the Silanised-treated glass fibers and manually fabricated two twisted soft wire retainers. A hazard ratio of retainer detachment was two times smaller in case of use of new kind of retention. The risk of failure was approximately 50% less for TDW retainers compared to FSW retainers, though it was not statistically significant.
**Lee and Mills 2009** [39]	Case – Control Clinical Trial	Comparison of the failure rate of fixed orthodontic retention between 2 different wires (stand. SS multistranded wire to innovative SS black Australian wire in V-loop design)	300 patients153 SSW (9–60 yrs. Old; 65% W, 35% M)147 VL (9–58 yrs. Old; 68%W, 32% M)	.0175-in stainless steel multi-stranded wire placed as a straightVs..016-in stainless steel black Australian wire placed in a V-loop design	Debonding rate within 6 months observation period (checked monthly)	The detachment rates were 14.3% for the V-loop design and 12.4% for the straight wire retainer. However, the differences remain statistically insignificant. No wire fracture of deformation was observed.
**Taner and Aksu 2012** [40]	Case – Control Study	Comparison of failure rate of flexible, braided rectangular bonded lingual retainers, discover differences in directly and indirectly bonded patients and the distribution of failures in time	66 patients (52 W, 14 M) divided in unequal groups	An eight-braided, flattened, stainless steel dead soft wire (Bond-a-Braid, 0.016 × 0.022 in.; Reliance Orthodontic Products, Itasca, Illinois, USA) bonded directly Vs.Bonded indirectly	Survival rate, distribution of failures in time within 2 years period (checked every 6 months)	Through the whole follow-up period, 25/66 had experienced failures. The failure rate was 46.9% with retainers bonded with the direct method and 29.4% with the indirect method. The highest failure rate was seen in the first month, a total of 24 failures occurred in 13 patients. The highest rate was 33.3% for the lower right central incisor. The lowest failure rate was observed in the fifth month, a total of three failures in two patients. From the total of 25 patients who had failures, 7 had repeated bond failures. The method of bonding does not affect the success of retention.
**Renkema et al. 2011** [41]	Retrospective cohort study	Assessment of the long-term effectiveness of FSW canine-to-canine lingual retainers in maintaining the alignment of the mandibular anterior teeth after orthodontic treatment	221 patients (75 W, 146 M)	Position of tooth before treatment Vs. Position of tooth after treatment Vs. 2 years after treatment Vs. 5 years after treatment obtained with FSW retainer (0.0195-in, 3-strand, heat-treated twist wire, Wildcat, GAC International, Bohemia, NY)	Little’s index within 5 years period (checked every year)	At T2, the irregularity index was stable in 93.7% of the patients; at T5, it was stable in 90.5%. According to Little’s index, at T0 the alignment of the mandibular front teeth was very good (irregularity index\1.00 mm) in 97.7% of the patients; at T2 and T5 the percentages were 95.0 and 93.7%, respectively. 32.2% of our patients experienced retainer failures. In only 1 patient, the retainer was broken. The bonding failure rate (ie, bonding failures per year) was higher during the first 2 years after treatment (32.0% from T0 to T2 and 17.6% from T0 to T5). Retainer failures were obtained from the patient files.
**Farronato et al. 2014** [42]	Retrospective cohort study	Evaluation of the long term results of fixed retention with FRC lingual retainers	119 patients, 15 with retainers in both arches (134 retainers)	Influence of gender, patient age and retainer location on survival.	Survival rate within ca. 39.9 months (median 40.7 months, SD 13.3 months) (checked every 6 months)	In total, fracture or delamination of the composite was recorded in 25 FRC retainers (18.7%). The incidence of relative failures was lower for the mandibular than for the maxillary retainers. The researchers found no correlation between any of the personal characteristics and survivial rate. Regrettably, however, the article lacks a summary table of the personal characteristics and their possible impact.
**Kocher et al. 2019** [43]	Retrospective cohort study	Comparison of failure rate of two types of fixed orthodontic retainer	88 patients	.016″ × .022″ braided SS (ORMCO) bonded to all six mandibular anterior teeth in mandible and four incisors in maxillaVs..027″ round TMA wire bonded to canines only (ORMCO)	Failure rate and type of failure within 10 to 15 years afterorthodontic treatment	The original retainer was still in situ 10–15 years after debonding for 87 patients (98.9%) in the mandible and for 80 patients (97.6%) in the maxilla. No failure of any type was observed in the mandible for 19 (40.4%) patients fitted with the .016″ × .022″ braided SS retainers and 25 (61%) with the .027″ round TMA retainers. The only significant predictor for survival was the type of retainer. (higher in SS group) In bothjaws, the most frequent first failure was composite damage(mandible 22.7%; maxilla 13.4%) followed by detachment(mandible 19.3%; maxilla 7.3%). Six events 6.8% ofloss of the whole retainer with need for replacement in the mandible were observed. No retainer fractured.

### Quality assessment

Due to the fact that three types of studies were qualified for the review, three different specific scales from the Cochrane Collaboration Handbook were used. The evaluation of RCTs is presented in Table 2 in a descriptive form, while the evaluation of the included CCs and cohort studies in numerical form, successively in Tables 3 and 4.

**Table 2:** Evaluation of included studies according to revised tool for assessing risk of bias in randomized trials (RoB 2)

	**Bovali et al. 2014** [24]	**Gunay et al. 2016** [25]	**Bolla et al. 2011** [26]	**Årtun et al. 1997** [27]	**Nagani et al. 2020** [28]	**Bazargani et al. 2012** [29]
Random sequence generation	LOW	SOME CONCERNS	SOME CONCERNS	HIGH	LOW	LOW
Allocation concealment	LOW	LOW	HIGH	LOW	LOW	SOME CONCERNS
Blinding of participants and personnel	HIGH	HIGH	HIGH	HIGH	HIGH	HIGH
Blinding of outcome assessment	LOW	HIGH	HIGH	HIGH	HIGH	LOW
Incomplete outcome data	LOW	LOW	LOW	LOW	LOW	LOW
Selective reporting	LOW	SOME CONCERNS	LOW	HIGH	LOW	LOW
Other bias (why)	Indirect reteiners were bonded with chemical curing, direct ones – with light curing, which may both influence chairtime and survival rate.	Extraction had longer reteiners (to premolars)	Uneven no. of participants study groups. Different bonding materials were used.	Clinical procedures were performed by different clinicians. The study group has been expanded from an earlier study.	In case if detachment of retainer was noticed during the visit, the subject was allowed to approach immediately for repair and bond failure was recorded in the upcoming visit.	NONE
Risk of bias judgement	LOW	SOME CONCERNS	HIGH	HIGH	SOME CONCERNS	LOW
	**Salehi et al. 2013** [30]	**Arash et al. 2020** [31]	**Scribante et al. 2011** [32]	**Rose et al. 2002** [33]	**Gelin et al. 2020** [34]	**Kartal et al. 2020** [35]
Random sequence generation	LOW	HIGH	SOME CONCERNS	SOME CONCERNS	LOW	LOW
Allocation concealment	LOW	SOME CONCERNS	SOME CONCERNS	SOME CONCERNS	LOW	LOW
Blinding of participants and personnel	HIGH	HIGH	HIGH	HIGH	HIGH	HIGH
Blinding of outcome assessment	SOME CONCERNS	SOME CONCERNS	HIGH	SOME CONCERNS	SOME CONCERNS	HIGH
Incomplete outcome data	LOW	SOME CONCERNS	LOW	LOW	LOW	LOW
Selective reporting	LOW	SOME CONCERNS	SOME CONCERNS	SOME CONCERNS	LOW	LOW
Other bias (why)	The study included patients during growth period.	The study included patients during growth period. Regression analysis was performed for statistically non-significant results.	No sample size calculation.	The study included patients during growth period. No data concerning drop-out is reported.	NONE	Patient’s age was not provided.
Risk of bias judgement	SOME CONCERNS	HIGH	HIGH	HIGH	LOW	LOW
	**Scribante et al. 2020** [36]	**Sfondrini et al. 2014** [37]	**Sobouti et al. 2016** [38]			
Random sequence generation	SOME CONCERNS	LOW	LOW			
Allocation concealment	LOW	LOW	LOW			
Blinding of participants and personnel	HIGH	HIGH	HIGH			
Blinding of outcome assessment	LOW	SOME CONCERNS	SOME CONCERNS			
Incomplete outcome data	LOW	LOW	LOW			
Selective reporting	LOW	LOW	LOW			
Other bias (why)	NONE	NONE				
Risk of bias judgement	LOW	LOW	SOME CONCERNS

**Table 3:** Newcastle-Ottawa Quality Assessment Form for Case-control Studies

Study		Lee and Mills 2009 [39]	Taner and Aksu 2012 [40]
**Selection**	Is the case definition adequate?	1	1
	Representativeness of the cases	0 – not described properly	0 – not described properly
	Selection of Controls	1	1
	Definition of Controls	1	1
**Comparability**	Comparability of cases and controls on the basis of the design or analysis	1	2
		The procedures of retainer bonding and follow – up were standardized. However, in some cases, not strictly defined, the procedure was modified. The number of teeth bonded to retainer differed in induvial patients.	The procedures of retainer bonding and follow – up were standardized. The same wire, adhesive and light curing unit were used in both groups. However, the additional chemical adhesive was used in indirect bonding group The patients were randomly assigned to the groups.
**Outcome**	Ascertainment of exposure	1	1
	Same method of ascertainment for cases and controls	1	1
	Non-Response rate	1	1
**Total**		7	8

**Table 4:** Newcastle-Ottawa Quality Assessment Form for Cohort Studies

Study		Renkema et al. 2011 [41]	Farronato et al. 2014 [42]	Kocher et al. 2019 [43]
**Selection**	Representativeness of the exposed cohort	1	0 – not properly described	1
	Selection of the non-exposed cohort	0	0	1
	Ascertainment of exposure	1	1	1
	Demonstration that outcome of interest was not present at start of study	1	1	1
**Comparability**	Comparability of cohorts on the basis of the design or analysis controlled for confounders	0	0	2
		There was only one cohort group, which was evaluated referring to different factors.	There was only one cohort group, which was evaluated referring to different factors.	The data collection and clinical evaluation were the same.
**Outcome**	Assessment of outcome	1	1	1
	Was follow-up long enough for outcomes to occur	1	1	1
	Adequacy of follow-up of cohorts	1	1	1
**Total=**		**6**	**5**	**9**

### Failures of fixed retainers

The failure rates in studies included ranged from 7.3% in the study by Kocher et al. [43] to 50% in the study by Rose et al. 2002 [33]. Patients, who once had the retainer detach, are at risk of repeated failure. Retention failure occurs more frequently in the maxilla than in the mandible. Concerning bonding failures statistically significant differences concerning failure rates, discoloration and calculus accumulation [29] have been reported between retainers bonded with additional use of a bonding agent versus retainers bonded with a composite material only [29, 36]. Adhesive failure (with no retained composite material on enamel surface) is the most common type of bond failure observed in fixed lingual retainers [28, 42] Concerning the stability of dental alignment, irregularity index increases with time despite the presence of bonded retainers [25, 27, 41]. No difference has been confirmed as far as type of wire and splint was concerned. Referring to composite material used for retainer bonding, one study has been included proving less failures with Transbond XT versus Filtek Supreme XTE (flowable) [36]. It must be noted, that retainers bonded to the surface of all adherent teeth were superior concerning stability of alignment to retainers bonded to two marginal teeth only [27].

The novelty - customized CAD/CAM (computer-aided design/computer-aided manufacture) nitinol retainer provides neither superior stability nor lesser gingival influence compared to classical twisted stainless-steel wire retainer [34, 35]. Referring to the bonding procedure: direct vs. indirect the only difference is shorter chairtime [24, 40].

### Meta-analysis

Opinions on the superiority of retention with FRC (fiber reinforced composite) or multistranded SS wire are contradictory in the included studies. Therefore, it seems pertinent to carry out a meta-analysis of failure rates among the included studies that compare the retention efficiency of FRC and the 0.0175″ stainless steel wire. There were 7 included studies in meta-analysis. Total sample size of all included studies is 503 patients and 516 reteiners. The data used to extracted and perform meta-analysis is shown in Table 5.

**Table 5:** Data included in meta-analysis

Author, year of publication	FRC failure rate	0.0175″ Multistranded SS Wire failure rate	Follow – up (years)	Total sample size (patients)	Total sample size (reteiners)
Bolla et al. 2011 [26]	11/48 retainers	17/50 retainers	6	85	98
Nagani et al. 2020 [28]	67/156 teeth	49/156 teeth	1	52	52
Salehi et al. 2013 [30]	34/68 retainers	27/74 retainers	1,5	142	142
Scribante et al. 2011 [32]	13/90 teeth	23/102 teeth	1	34	34
Rose et al. 2002 [33]	5/10 retainers	1/10 retainers	2	20	20
Sfondrini et al. 2014 [37]	27/240 teeth	50/282 teeth	1	87	87
Sobouti et al. 2016 [38]	15/42 retainers	11/41 retainers	2	83	83

**Fig. 2:**
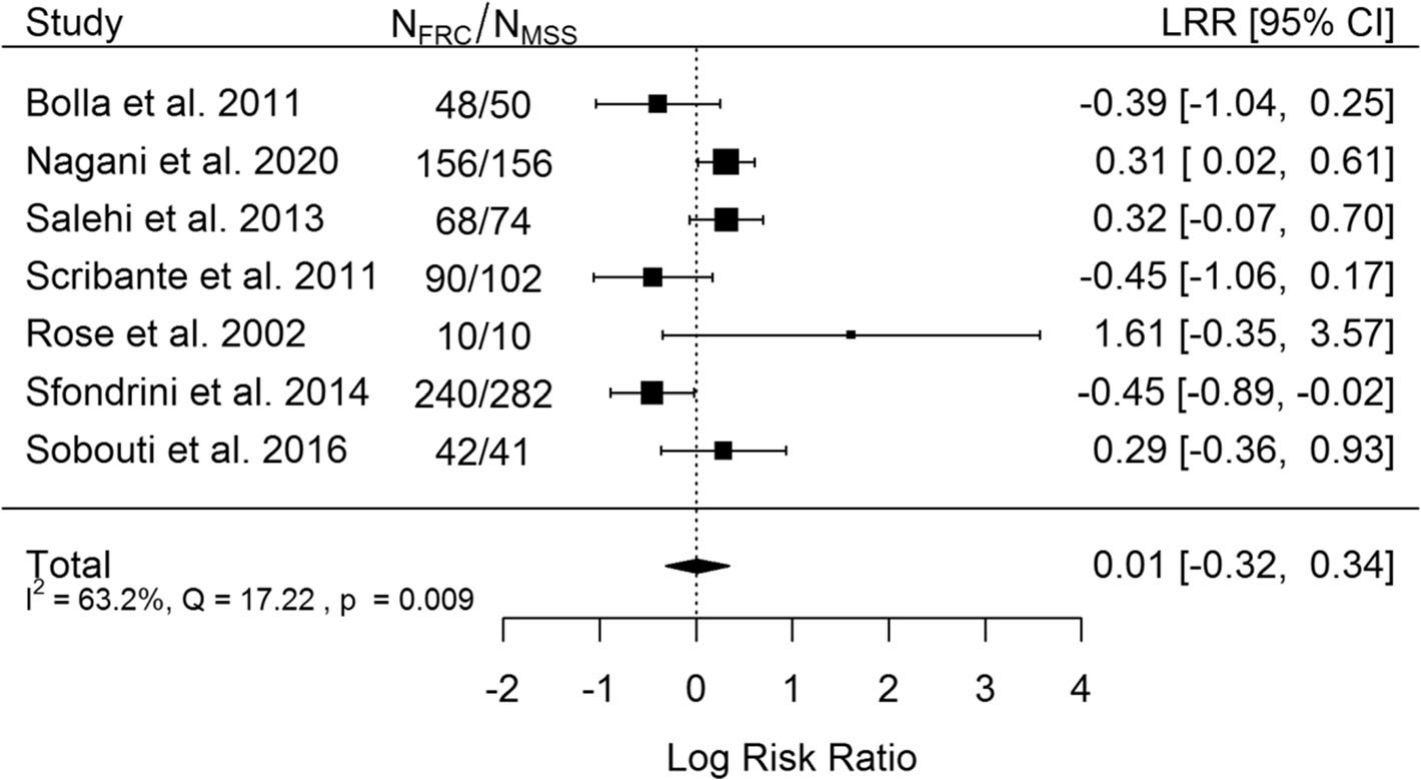
Forrest plot

The results are shown on Fig. 2. Positive value of LRR indicates greater risk of FRC usage, negative – of MSS usage. Three studies showed very consistently the advantage of FRC and three - equally consistent with the advantage of multistranded steel wire. The study by Rose et al. reports results contradictory to all the other studies included in the meta-analysis. However, it does not have a large impact on the results of the meta-analysis, due to the small size of the studied sample. As it can be seen from the results of the calculations, there is no statistically significant difference between the type of material used and the possible risk of failure.

**Fig. 3:**
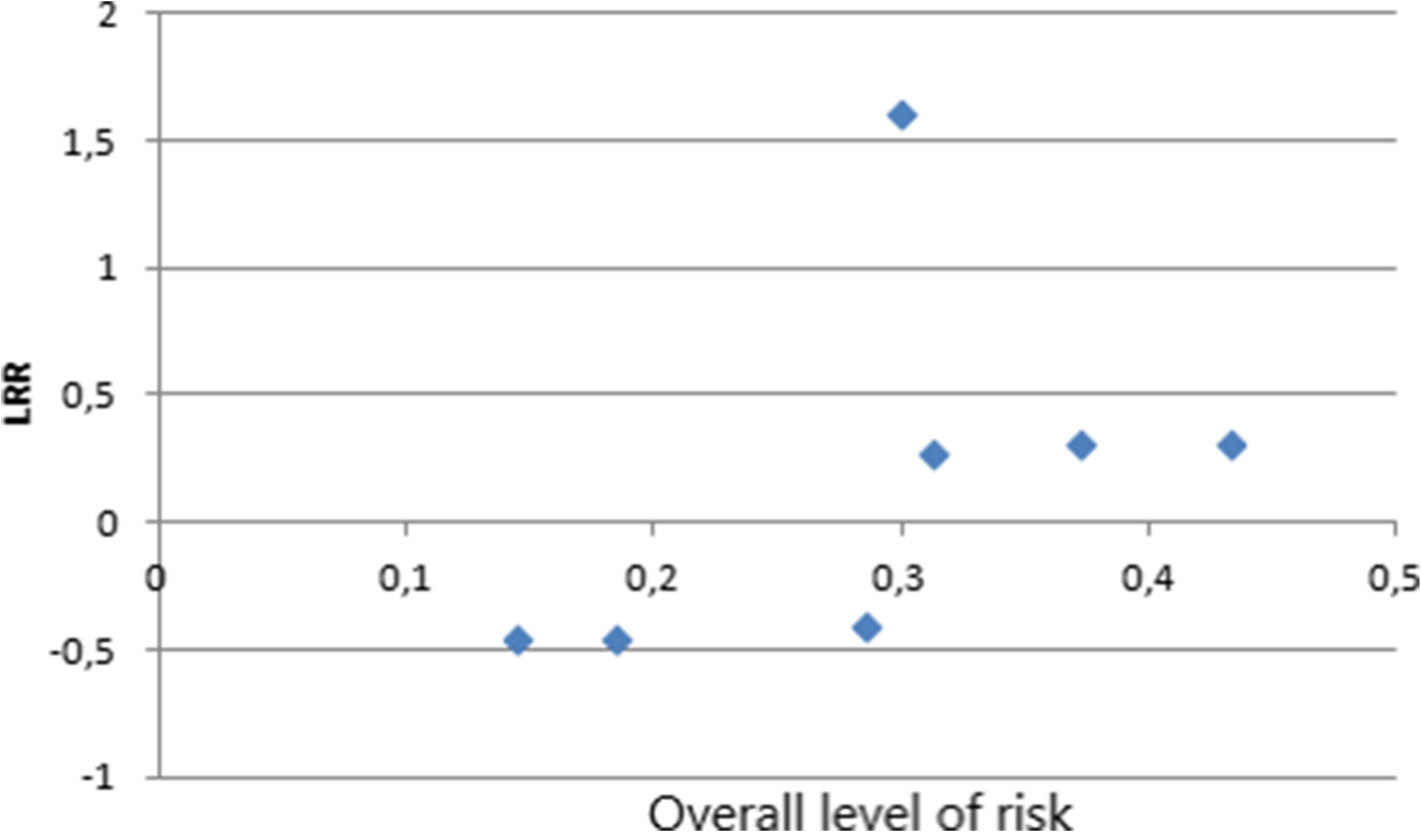
LRR ratio to overall level of risk

The higher the risk of FRC retention failure in the studies the higher the overall level of risk. (Fig. 3). This indicates that in the studies where failure occurred much more often than in the others, there was a much greater risk of retention failure while using FRC than while using SS wire. This indicates that FRC retention is definitely more sensitive to operator skills, and with incorrect technique of bonding, the possibility of failure events is much higher.

**Fig. 4:**
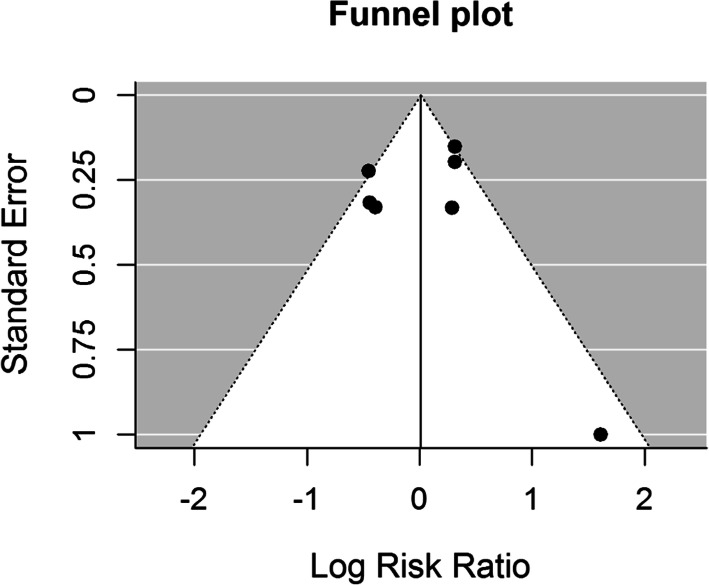
Funnel plot

The type of wire used has an insignificant (*p* = 0.868) effect size. Results of the studies included are inconsistent – heterogeneity is significant (*p* = 0.009), 63.2% of the variability come from heterogeneity. Funnel plot (Fig. 4) does not reveal publication bias.

Due to the fact that the included studies differed in the units in which the failure rate is expressed (no. of detached reteiners and no. of detached teeth), it was examined whether there were significant differences between this group of studies.

**Fig. 5:**
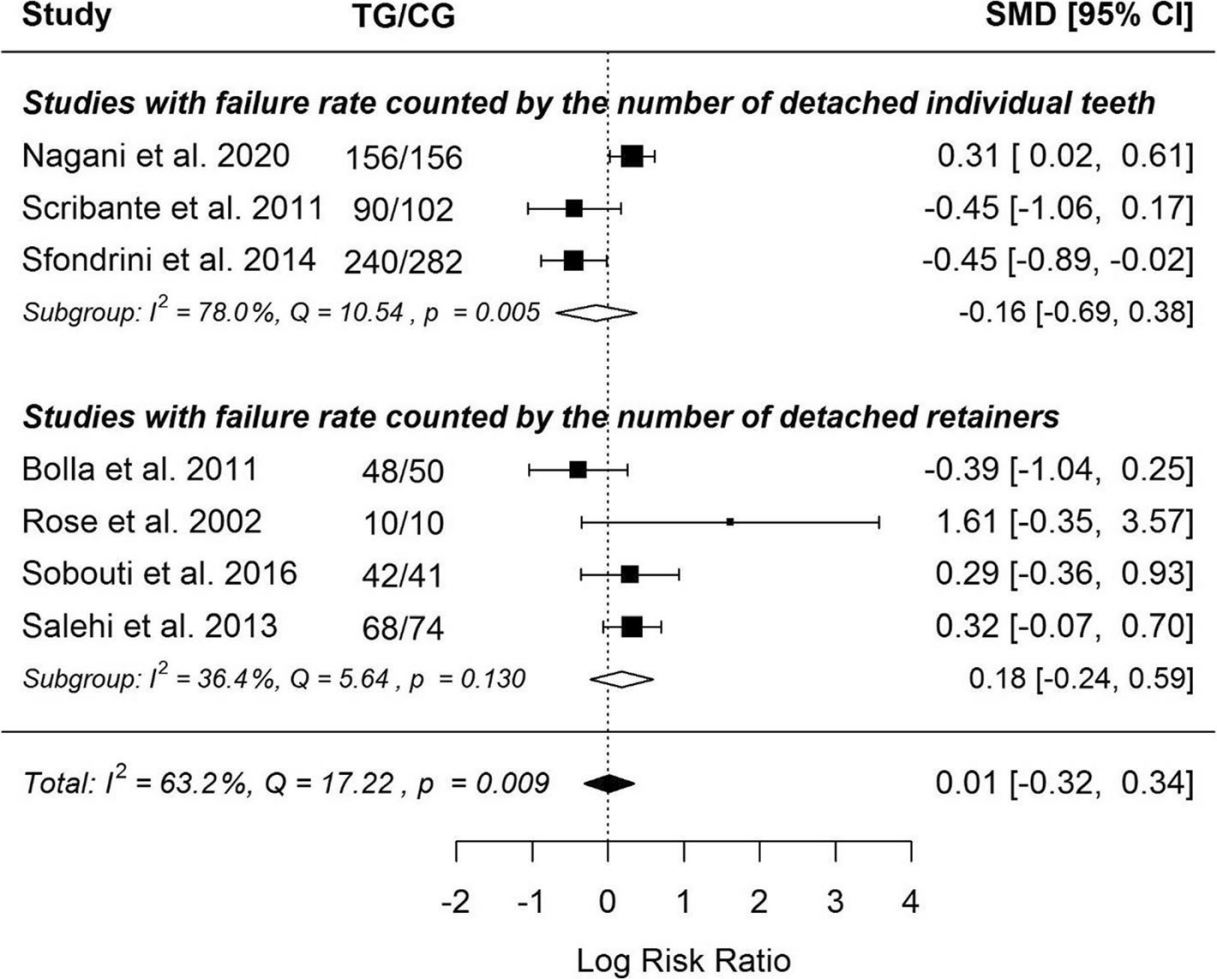
Forrest plot considering 2 groups of studies with different units of failure rate

In the group of studies with failure rate counted by the number of detached individual teeth used wire has small insignificant negative effect size, while in the group of studies with failure rate counted by the number of detached retainers – small positive effect size. Study results were inconsistent in the first group – heterogeneity was significant (*p* = 0.005), 78.0% of the variability come from heterogeneity. However, despite the split, there were still no significant differences between the FRC and MSS groups. This means that the “unit of measure” of failure did not affect the overall conclusions coming from the meta-analysis (Fig. 5).

**Fig. 6:**
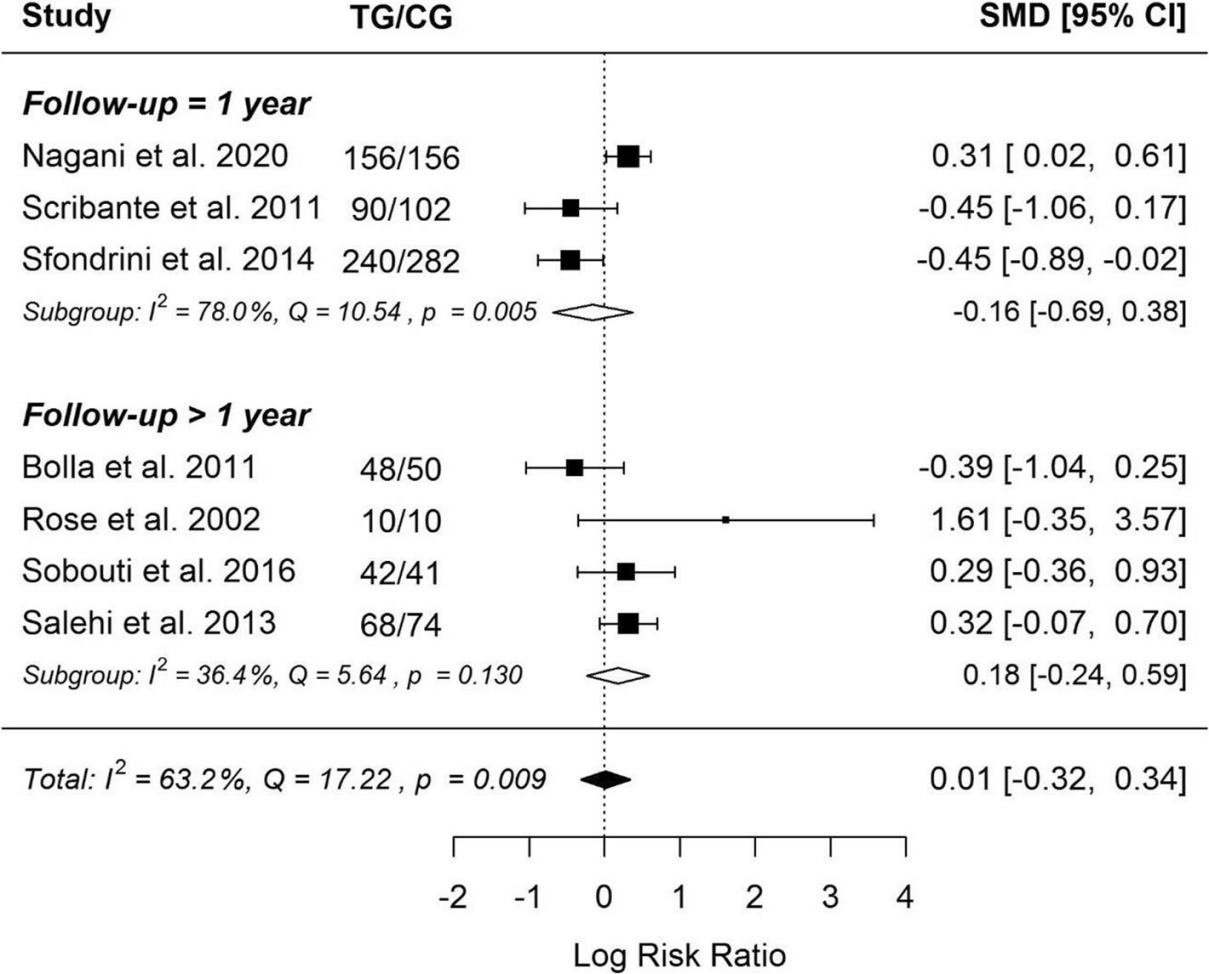
Forrest plot considering 2 groups of studies with different duration of follow- up

It seems also extremely interesting whether the follow-up time was not a factor that may differentiate the effectiveness of the compared types of retention. Therefore, it was concluded that it is worth sub-classifying the included studies into two groups – short-term (up to 12 months) and long-term follow-up (more than 12 months). The shortest follow-up in the included studies was 1 year. That is why 1 year has therefore become the dividing line between short-term and long-term research (Fig. 6).

However, also in this part of the study, there was no statistically significant difference between the two groups, which strongly confirms the statement made on collective meta-analysis. The studies included in the entire meta-analysis were highly heterogeneous, but it results from the specificity of the treatment in the retention phase – clinical approach was diverse in various studies.

## Discussion

This systematic review aimed to set out the viable evidence on possible factors affecting the failure of fixed orthodontic retention, using both qualitative and quantitative synthesis.

The follow – up period in included RCTs ranged from 6 months in the study by Lee and Mills [39] to 24 months by Sobouti et al. 2016 [38] and in all of RCTs patients has been checked monthly. In cohort studies follow – up period lasted from 40 months to 15 years, while in case-control studies it ranged from 6 months to 2 years with different frequency of check-ups. It is worth mentioning, that the failures tend to occur mostly within 2 years after retainer placement, [41] mostly in the first 3 months period, [24, 33, 40] or within first 6 months, when after this time occurred first check-up [42].

According to the literature, failure of a fixed retention may result from detachment between wire and composite, breaking the interface between adhesive and enamel, wire deformation or untwisting (round wires) [44] or wire fracture within approximal surface [45]. The critical factors influencing a successful retainer bonding are: clean enamel surface to be bonded, dry field and avoiding occlusal interference [46]. The present study shows that breaking the interface between wire and composite or wire deformation are not prevalent causes of failures in fixed retention. The same refers to wire untwisting - published in case reports [44, 47] and in-vitro studies [48, 49] – it did not occur in any of the studies meeting the inclusion criteria for a systematic review. On the other side, breaking the interface between composite and enamel is a very important cause for a failure. It is evident that maximum effort should thus be made to improve the bonding procedure. The present study confirmed the importance of the skills of the personnel to perform the bonding procedure. Moreover, the use of a bonding agent proved to improve bonding efficacy.

Concerning wire fracture, from the present study, it is a factor of failures, but not the one of the highest importance. Contrary conclusions refer to FRC which rather tends to break than to debond.

In the included studies, no clear effect of the wire used (SS wire, NiTi) on the patient’s periodontal condition was found [27, 34, 35]. Either, in the literature, no detrimental effect of the presence of a retention wire on the health of periodontal tissues was found, indicating a greater risk of periodontitis in the fixed retention obtained with FRC [50–52]. Interestingly, the positioning of the retainer more coronally or gingivally should not affect the occurrence of symptoms of periodontal tissues, either [53]. The most important factor influencing the presence of plaque, calculus or inflammation is the patient’s awareness and daily hygiene, that is, factors that would always present better in clinical trial conditions than in patients who do not undergo specific, frequent control [50]. Bonding to enamel proved to be critical for the success of permanent retention. It is obvious that bonding strength is influenced by the etching procedure [54, 55]. In the studies included, etching times declared ranged from 15 s to 60s, the most prevalent time was 30s. Moreover, the concentrations of phosphoric acid were different, from 32 to 37%. No detailed description of the rinsing time is provided in any of the studies included, most authors write that the etching agent was “rinsed thoroughly”. Eventual effect of the etching or rinsing time on debonding rates could be a subject of future studies, that could improve the existing knowledge and allow to create clinical recommendations.

A phenomenon that most influences the risk of bias in randomized clinical trials is the nature of the process itself. It is impossible to perform blinding on the staff or on the patient. Both are aware of the bonding fixed retainer process taking place. In the case of studies verifying patient satisfaction, patient itself was able to distinguish the FRC or CAD / CAM retainer from the standard retainer due to its appearance or method of application. Most RCTs did not provide an objective evaluation of the clinical results of the applied solutions by a different investigator than the one who was not involved in the retention bonding, either. This could be a simple solution to implement, and certainly reducing the risk of bias. The vast majority of studies demonstrate no problems with introducing and describing the processes of randomization and allocation in patients selection for the study groups as well as in outcome data presentation. A possible risk of selective reporting was found in several studies [25, 27, 31–33]. However, it should be pointed out, that there were other risks of bias. The most prevalent were: the use of different bonding agents in different study groups (Bovali and Bolla) [24, 26] and including patients during growth (under 18th years of age). Growth affects the stability of retention, thus the number of young people in the distribution of the study group could have significantly influenced the results of the research. (Sobouti, Kartal, Rose, Salehi, Arash) [30, 31, 33, 35, 37]. Tooth movement resulting from ageing occurs in all subjects, disregarding the positive or negative history of orthodontic treatment. In most of the studies included in review of Schubert et al., young patients showed faster orthodontic movement in the first phase of treatment and more pronounced cytokine levels [56].

The clinical-control studies did not present high level of risk of the bias. However, in both cases, the criteria for including patients in the study were not adequately described [39, 40]. Doubts arise on the representativeness of the study groups, and thus the adequacy of the study results to the general population. Lee and Millis sometimes altered the procedure of retainer bonding in an inaccurately defined number of patients, which could have an impact on their results [39].

The included cohort studies were at greatest risk of bias. In the studies of Renkema et al. [41] and Farronato et al., [42] there was only one cohort group, which was evaluated referring to different personal factors. Additionally, Farronato et al. [42] did not adequately provide inclusion criteria to the cohort. In contrast, the studies by Kocher et al. [43] were designed, conducted and described in a model way for this type of research. This systematic review has some limitations due to several factors. First of all, this study is limited by the heterogeneity of design between included studies, different types of wire used, different outcomes measured and elements on which some studies focused on. Many studies have also included developmental follow-up on patients. Growth inevitably has an impact on the outcome stability in the retention phase. Another limitation of this systematic review is the period of follow-up and different frequency of check-ups in the included studies. The vast majority of patients came for control visits every month. However, there are studies in which the retention status was tested every few months, 6 months or even every year. From the results of observations from monthly surveys, we are able to conclude that such a rare observation causes many processes in the oral cavity to be missed at the beginning of the retention phase. In meta-analysis, as failure rate, fractures and debonding were introduced together as failures. Breakage is caused by stiffness of the material and deboning is associated with its elasticity. Thus, both this type of failures are material-depended, that is it seemed reasonable to put them in the one pool.

Thus, the available scientific evidence did not support a superior quality of any wire type or fiber splint, regarding failure rate or stability of the alignment. However, fiber splints break more often, whereas flexible spiral wires are more likely to debond [26, 30, 33, 43]. Round TMA (Titanium-Molybdenum alloy) .027″ wire and braided SS (Stainless Steel) .016″ × .022″ did not tend to break within 15 years of follow-up. However, stainless steel wires were more prone to detach than TMA [43]. It has been proved, that patients consider fiber splint wires as more aesthetic and comfortable than flexible spiral SS wire [40]. Concerning the bonding procedure, it has been proved that indirect bonding is less chair-time consuming. As far as bonding materials are concerned, the use of bonding resin significantly reduces failure rate. A single study reported less failures with Transbond XT versus flowable Filtek Supreme XTE.

## Conclusions

1. The follow-up periods in RCTs and in case-control studies ranged from 6 months to 2 years; in cohort studies - from 3 to 15 years.
2. No retainer is proved to guarantee a perfect stability of dental alignment.
3. The failure rate ranges from 7.3 to 50%, mostly occurs in the first 3 to 6 months of retention, more frequently in the maxilla than in the mandible.
4. The retainer should be bonded to all the adherent teeth, preferably with the additional use of the bonding agent.
5. No wire or fiber splint present superior characteristics concerning failure rate.
6. In the first 6 months after retainer bonding the patient should be under frequent control of retention status.
7. Indirect retainer bonding is associated with a shorter chairtime.
8. FRC retention is definitely more sensitive to operator skills, and with imperfect technique of bonding, the possibility of failure is much higher

## Supplementary Information

**Additional file 1.**

**Additional file 2.**

## Data Availability

Not applicable.

## Abbreviations

SS: Stainless steel
TMA: Titanium-Molybdenum Alloy
FRC: Fiber reinforced composite
RCT: Randomized clinical trial
RCCT: Randomized controlled clinical trial
CCS: Case-control study
CAD/CAM: Computer-aided design/computer-aided manufacture
LRR: Log Risk Ratio
